# Doublecortin May Play a Role in Defining Chondrocyte Phenotype

**DOI:** 10.3390/ijms15046941

**Published:** 2014-04-22

**Authors:** Dongxia Ge, Qing-Song Zhang, Jovanny Zabaleta, Qiuyang Zhang, Sen Liu, Brendan Reiser, Bruce A. Bunnell, Stephen E. Braun, Michael J. O’Brien, Felix H. Savoie, Zongbing You

**Affiliations:** 1 Department of Structural and Cellular Biology, Tulane Cancer Center, Louisiana Cancer Research Consortium, Tulane Center for Aging and Tulane Center for Stem Cell Research and Regenerative Medicine, Tulane University Health Sciences Center, New Orleans, LA 70112, USA; E-Mails: gedongx@gmail.com (D.G.); qingsong.chang@gmail.com (Q.-S.Z.); qzhang3@tulane.edu (Q.Z.); senliu@yahoo.com (S.L.); brendan.reiser@gmail.com (B.R.); 2 Department of Orthopaedic Surgery, Pu Ai Hospital of Tongji Medical College, Huazhong University of Science and Technology, Wuhan 430033, China; 3 Department of Pediatrics and Stanley S. Scott Cancer Center, Louisiana State University Health Sciences Center, New Orleans, LA 70112, USA; E-Mail: jzabal@lsuhsc.edu; 4 Department of Pharmacology, Tulane Center for Stem Cell Research and Regenerative Medicine and Division of Regenerative Medicine of Tulane National Primate Research Center, Tulane University Health Sciences Center, New Orleans, LA 70112, USA; E-Mails: bbunnell@tulane.edu (B.A.B); sbraun@tulane.edu (S.E.B.); 5 Department of Orthopaedic Surgery and Tulane Institute of Sports Medicine, Tulane University School of Medicine, New Orleans, LA 70112, USA; E-Mails: michaelobrien76@gmail.com (M.J.O.); fsavoie@tulane.edu (F.H.S.)

**Keywords:** articular cartilage, chondrocytes, doublecortin, *DCX*

## Abstract

Embryonic development of articular cartilage has not been well understood and the role of doublecortin (*DCX*) in determination of chondrocyte phenotype is unknown. Here, we use a *DCX* promoter-driven *eGFP* reporter mouse model to study the dynamic gene expression profiles in mouse embryonic handplates at E12.5 to E13.5 when the condensed mesenchymal cells differentiate into either endochondral chondrocytes or joint interzone cells. Illumina microarray analysis identified a variety of genes that were expressed differentially in the different regions of mouse handplate. The unique expression patterns of many genes were revealed. *Cytl1* and *3110032G18RIK* were highly expressed in the proximal region of E12.5 handplate and the carpal region of E13.5 handplate, whereas *Olfr538*, *Kctd15*, and *Cited1* were highly expressed in the distal region of E12.5 and the metacarpal region of E13.5 handplates. There was an increasing gradient of *Hrc* expression in the proximal to distal direction in E13.5 handplate. Furthermore, when human DCX protein was expressed in human adipose stem cells, collagen II was decreased while aggrecan, matrilin 2, and *GDF5* were increased during the 14-day pellet culture. These findings suggest that *DCX* may play a role in defining chondrocyte phenotype.

## 1. Introduction

Diseases of articular joints, such as osteoarthritis, cause pain and impaired mobility. It is estimated that 24.3 million American adults have osteoarthritis [1]. The current clinical treatments, other than total joint replacement, do not change the course of osteoarthritis. Regenerative medicine, including tissue engineering, offers exciting opportunities to restore functional articular cartilage. However, currently the tissue-engineered cartilages behave like physeal or endochondral cartilages that undergo premature hypertrophy, unlike the stable articular cartilage that lasts a lifetime [2]. The current tissue engineering of cartilage follows a paradigm of high-density cell culture such as pellet culture, micromass culture, or high-density culture in certain matrix scaffolds. This paradigm essentially mimics embryonic development of skeletal anlage (*i.e.*, endochondral cartilage), rather than articular cartilage. Although endochondral and articular cartilages are both hyaline cartilages, they differ significantly [3]. Embryonic development of articular cartilage has not been well understood. This lack of complete understanding of articular chondrocyte phenotype establishment is a problem for the field of articular cartilage tissue engineering/regeneration.

It is well recognized that the mechanism of regeneration recapitulates the mechanism of embryonic development [4,5]. Therefore, it is critical to understand the determinants of chondrocyte phenotype during embryonic chondrogenesis. In mouse embryonic limb buds, the mesenchymal cells appear homogeneous at embryonic stages of 9.5 to 11.5 days postcoitus (*i.e.*, E9.5 to E11.5). Mesenchymal condensation occurs in the limb buds at E12.5, without any signs of joint interzones—presumptive sites of articular joints [6]. At E13.5, joint interzones appear in the proximal to distal order [7], and the long bone anlagen intervening between the joint interzones become cartilaginous with endochondral chondrocytes residing within the anlagen [8]. Joint interzone can be recognized by postmortem histologic staining or LacZ staining in mice with *Gdf5*-*cre*-driven *LacZ* expression [8]. Recently, live imaging of joint interzones became possible when doublecortin reporter mice were developed [9]. Doublecortin (*DCX*) is a gene located on chromosome Xq22.3-Xq23, encoding a microtubule-binding protein that is expressed in migrating and differentiating neurons [10–12]. We originally found that *DCX* is expressed in human and mouse articular chondrocytes, but not in endochondral chondrocytes, synovium, or cruciate ligaments [13]. Using two reporter mouse strains with *DCX* promoter-driven *LacZ* or enhanced green fluorescence protein (*eGFP*), we found that *DCX* is expressed in the mesenchymal cells in mouse embryonic limb buds, however, a population of mesenchymal cells maintain *DCX* expression when they differentiate into joint interzone cells and articular chondrocytes, whereas the other population of mesenchymal cells that differentiate into endochondral chondrocytes lose *DCX* expression [9]. The *DCX-EGFP* reporter mouse provides a unique tool to investigate the dynamic changes of chondrocyte phenotype *in vivo* or *ex vivo*.

## 2. Results and Discussion

### 2.1. The DCX-Positive Proximal and DCX-Negative Distal Regions of E12.5 Mouse Handplate Express Different Genes

**Figure 1.**
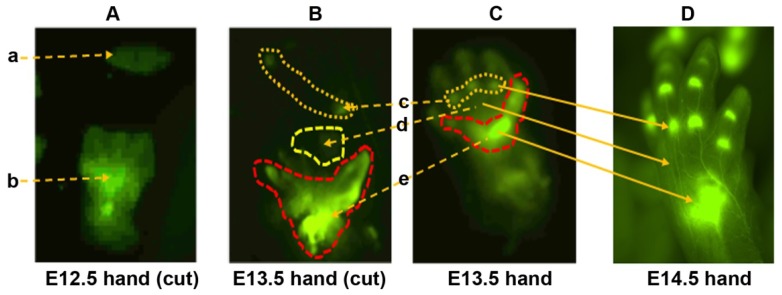
Illustration of dissection of mouse embryonic handplates. (a) the distal region (DCX-eGFP-negative); (b) the proximal region (DCX-eGFP-positive); (c) the metacarpal-phalange region (DCX-eGFP-positive); (d) the metacarpal region (DCX-eGFP-negative); (e) the carpal region (DCX-eGFP-positive). All photomicrographs were taken under an epifluorescence microscope with 6× (**A**); 1.3× (**B**) and (**C**); and 1× (**D**) original magnification.

Limbs develop in the proximal to distal order [7]. The regional differences are obvious morphologically. This study focused on mouse handplates at E12.5 to E13.5, because differentiation of the condensed mesenchymal cells into chondrocytes occurs during this period. Our previous study showed that the proximal region of E12.5 handplate expresses high levels of *DCX* as shown by eGFP signals, whereas the distal region is almost negative for eGFP signals except the faint signals in the digit rays [9]. Thus, we cut mouse handplates into proximal and distal regions based on eGFP signals under an epifluorescence microscope (Figure 1A). RNA was isolated from the proximal and distal tissues and Illumina microarray analysis was performed. Judging by a two-fold difference, we found that there were 34 genes with mRNA expression levels higher in the proximal region than the distal region, while there were 44 genes expressed at higher levels in the distal region than the proximal region (Tables 1 and S1). Many of these genes have never been studied in limb development. Delta-like 1 homolog (*Dlk1*, No. 1 in Table 1) was highly expressed in the proximal region of mouse handplate, where mesenchymal condensation occurs and chondrogenesis is ongoing. However, it has been shown that *Dlk1* inhibits *in vitro* chondrogenesis [14]. We speculate that the increased *Dlk1* level may be needed to antagonize other signals that drive chondrogenesis, so as chondrogenesis occurs in a controlled manner. *Cytokine-like 1* (*Cytl1*, No. 3 in Table 1) was a gene highly expressed in the proximal region of mouse handplate. Cytl1 is a secreted, cytokine-like factor that has chondrogenic effect via stimulation of sex determining region *Y-box 9* (*Sox9*) transcriptional activity [15]. The increased level of *Cytl1* in the proximal region possibly correlates with the earlier start of chondrogenesis in the proximal region than the distal region. However, a recent study showed that deletion of the *Cytl1* gene did not affect chondrogenesis or cartilage development [16]. In that study, *Cytl1*-null mice also showed normal endochondral ossification and long bone development. In addition, the ultrastructural features of matrix organization and chondrocyte morphology in articular cartilage were similar between wild-type and *Cytl1*-null mice. However, *Cytl1*-null mice were more sensitive to osteoarthritic (OA) cartilage destruction upon destabilization of the medial meniscus of mouse knee joints. Furthermore, the expression levels of Cytl1 were markedly decreased in OA cartilage of humans and experimental mice. Therefore, the authors of that study concluded that, rather than regulating cartilage and bone development, Cytl1 is required for the maintenance of cartilage homeostasis, and loss of Cytl1 function is associated with experimental OA cartilage destruction in mice [16]. Another gene, *paired related homeobox 2* (*Prrx2*, No. 19 in Table 1), was highly expressed in the distal region compared to the proximal region. It has been reported that *Prrx2* is highly expressed in undifferentiated mesenchymal cells and its expression decreases when the mesenchymal cells differentiate into chondrocytes [17]. Given that chondrogenesis proceeds from the proximal region to the distal region, it makes sense that the distal region with more undifferentiated mesenchymal cells expresses higher levels of *Prrx2*. Therefore, our results, at least for *Cytl1* and *Prrx2*, are consistent with the published literature.

### 2.2. The DCX-Positive and DCX-Negative Regions of E13.5 Mouse Handplate Express Different Genes

At E13.5 in mouse handplate, the condensed mesenchymal cells already differentiate into endochondral chondrocytes or joint interzone cells. The joint interzones are clearly shown by expression of eGFP signals in the *DCX-EGFP* mice [9]. Based on the principle of proximal to distal development, the joint interzones in the carpal region develop first, followed by the joint interzones between the metacarpal bones and phalangeal bones, and then the joint interzones between the phalangeal bones. Due to the gel-like physical property and small size of mouse handplate at E13.5, it is very difficult to dissect out individual joint interzones and cartilaginous anlagen. Thus, we cut the mouse handplate into three regions, namely, the carpal region (containing *DCX*-positive joint interzones), metacarpal region (containing *DCX*-negative metacarpal cartilaginous anlagen), and metacarpal-phalange region (containing *DCX*-positive joint interzones between the metacarpal bones and phalangeal bones) (Figure 1B,C). These regions are so designated as we previously observed that they correspond to the aforementioned regions at E14.5 when hand morphogenesis becomes very clear (Figure 1D and reference [9]).

Microarray analysis showed that there were only four genes that were expressed at higher levels in the carpal region than the metacarpal region (Tables 2 and S2). One of these genes is *Cytl1* (No. 2 in Table 2). Since *Cytl1* has chondrogenic effect and its expression is high in articular chondrocytes [18], it is reasonably expected that *Cytl1* level should be higher in the joint interzones of the carpal region than the cartilaginous anlagen in the metacarpal region, which is consistent with the expression pattern at E12.5. Microarray analysis also showed that there were 28 genes that were expressed at higher levels in the metacarpal region than the carpal region (Tables 2 and S2). Most of them have never been associated with chondrogenesis. *Frizzled homolog 10* (*Fzd10*, No. 8 in Table 2) is linked to Wnt signaling, and *Delta-like 2 homolog* (*Dlk2*, No. 10 in Table 2) inhibits NOTCH1 signaling. Both Wnt and NOTCH1 signaling pathways are known to play roles in chondrogenesis [19,20].

Microarray analysis showed that there were six genes that were expressed at higher levels in the metacarpal region than the metacarpal-phalange region (Table 3). One of them is *Delta-like 1 homolog* (*Dlk1*, No. 1 in Table 3). Dlk1 inhibits NOTCH signaling that inhibits chondrogenesis [20]. In contrast, there were 11 genes that were expressed at higher levels in the metacarpal-phalange region than the metacarpal region (Table 3). It appears that none of these genes has been studied in skeletal development.

### 2.3. Dynamic Gene Expression Profiles between the DCX-Positive Proximal Region of E12.5 Mouse Handplate and the DCX-Positive Carpal or Metacarpal-Phalange Region of E13.5 Mouse Handplate

Microarray analysis found that there were 62 genes with expression levels higher in the proximal region of E12.5 mouse handplate than the carpal region of E13.5 mouse handplate (Tables 4 and S3). Among them, *Cytl1* (No. 1 in Table 4), Dickkopf homolog 3 (*Dkk3*, No. 3 in Table 4), and *Dlk1* (No. 6 in Table 4) are known for their roles in chondrogenesis. In contrast, there were 47 genes that were expressed at higher levels in the carpal region of E13.5 mouse handplate than the proximal region of E12.5 mouse handplate (Tables 4 and S3). Most of these genes are not known for their roles in chondrogenesis, except *Fzd10* (No. 15 in Table 4) that may play a role in chondrogenesis through Wnt signaling [19].

Microarray analysis also showed that there were 79 genes that were expressed at higher levels in the proximal region of E12.5 mouse handplate than the metacarpal-phalange region of E13.5 mouse handplate (Tables 5 and S4). These genes include *Cytl1* (No. 3 in Table 5) and *Dkk3* (No. 9 in Table 5) that are known to play roles in chondrogenesis. However, the other genes have rarely been studied in chondrogenesis. On the other hand, there were 24 genes with expression levels higher in the metacarpal-phalange region of E13.5 mouse handplate than the proximal region of E12.5 mouse handplate (Tables 5 and S4). However, none of them has been studied in chondrogenesis.

**Figure 2.**
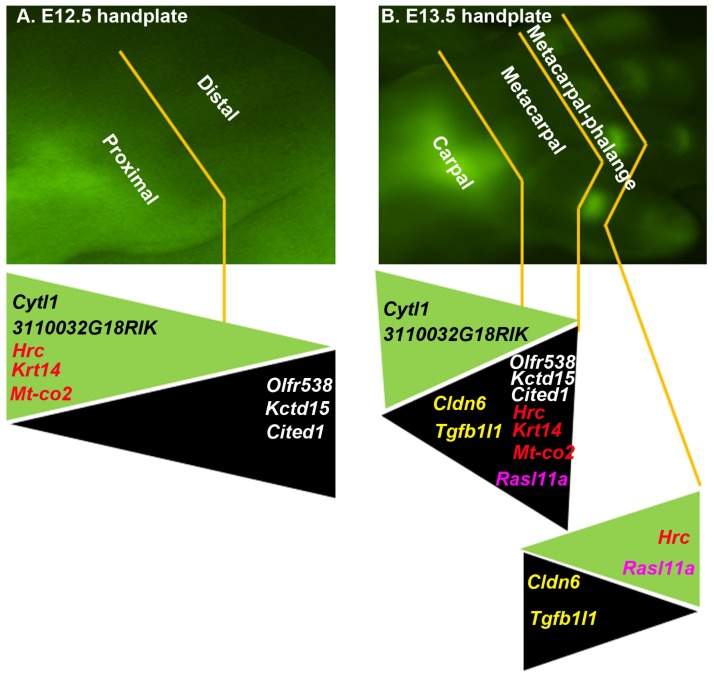
Comparison analysis of gene expression patterns between different regions. (**A**) E12.5 mouse handplate (original magnification, 6×); (**B**) E13.5 mouse handplate (original magnification, 1.3×). Selected genes are shown with their gene symbols color-coded and the ones with the same color are in comparison between the different regions. The genes are laid on colored triangles, the base of each triangle indicating higher levels of gene expression with the tip indicating lower levels of gene expression; green triangles indicate higher levels of gene expression in the DCX-eGFP-positive regions, whereas black triangles indicate higher levels of gene expression in the DCX-eGFP-negative regions.

In a comparison analysis of the above microarray data, we found some patterns of gene expression that are worth discussion. First, *Cytl1* and *3110032G18RIK* (also called *Fam101a*, *i.e.*, family with sequence similarity 101, member A) are consistently highly expressed in the *DCX*-positive proximal region of E12.5 mouse handplate and the *DCX*-positive carpal region of E13.5 mouse handplate, compared to the *DCX*-negative distal region of E12.5 mouse handplate and the *DCX*-negative metacarpal region of E13.5 mouse handplate (Figure 2A,B). On the opposite, Olfactory receptor 538 (*Olfr538*), Potassium channel tetramerisation domain containing 15 (*Kctd15*), and Cbp/p300-interacting transactivator with Glu/Asp-rich carboxy-terminal domain 1 (*Cited1*) are expressed at higher levels in the *DCX*-negative distal region of E12.5 mouse handplate and the *DCX*-negative metacarpal region of E13.5 mouse handplate than the *DCX*-positive proximal region of E12.5 mouse handplate and the *DCX*-positive carpal region of E13.5 mouse handplate (Figure 2A,B). These genes show a consistent expression pattern in the proximal to distal direction through E12.5 to E13.5; Second, several genes present a reverse expression pattern, including *Hrc*, *Krt14*, and *Mt*-*co2*. These genes are expressed at higher levels in the *DCX*-positive proximal region than the *DCX*-negative distal region of E12.5 mouse handplate (Figure 2A), however, their levels are lower in the *DCX*-positive carpal region than the *DCX*-negative metacarpal region of E13.5 mouse handplate (Figure 2B). Interestingly, the level of *Hrc* is higher in the *DCX*-positive metacarpal-phalange region than the *DCX*-negative metacarpal region of E13.5 mouse handplate (Figure 2B). These findings suggest that at E13.5, *Hrc* gene displays an expression pattern with increasing levels along the proximal-distal axis. *Rasl11a* gene also shows the similar pattern (Figure 2B); Third, *Claudin 6* (*Cldn6*) and *Transforming growth factor beta 1 induced transcript 1* (*Tgfb1I1*) genes are expressed at higher levels in the *DCX*-negative metacarpal region than the *DCX*-positive carpal region or metacarpal-phalange region of E13.5 mouse handplate (Figure 2B). Whether this expression pattern is linked to the difference between cartilaginous anlagen and joint interzone requires further investigation.

### 2.4. DCX Affects Expression of Genes Associated with Chondrocyte Phenotype

Our previous studies have demonstrated that *DCX* is expressed in the osteo-chondral mesenchymal precursor cells and its expression is maintained in joint interzone cells and articular chondrocytes [9,13]. Other investigators have also shown *DCX* expression in articular chondrocytes [21]. It has been recognized that the permanent cartilage (articular cartilage) expresses *DCX*, *growth differentiation factor 5* (*GDF5*), and versican, whereas the transient cartilage (skeletal anlagen or endochondral cartilage) expresses matrilin 1 [22]. However, the role of *DCX* in chondrogenesis has not been understood. Therefore, we studied whether consistent expression of low level of *DCX* in the mesenchymal stromal/stem cells (MSCs) would affect chondrocyte phenotype during chondrogenesis using a pellet culture model.

**Figure 3.**
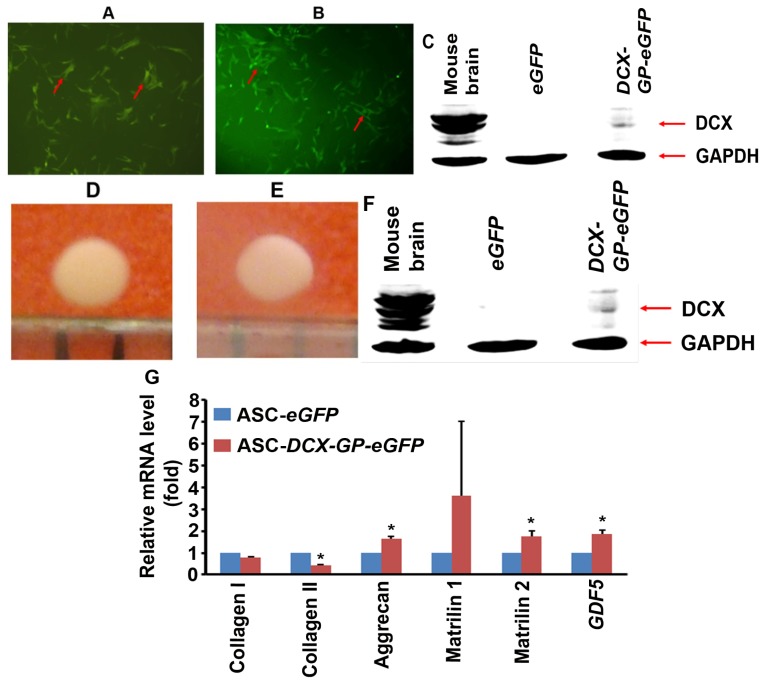
The effects of *DCX* expression on chondrocyte differentiation of human ASCs in pellet cultures. (**A**) and (**B**) Human ASCs transduced with HRST-*eGFP* (**A**) and HRST-*DCX-GP-eGFP* (**B**) lentiviruses and sorted by flow cytometry; arrows indicate eGFP-positive cells; original magnification, 200×; (**C**) Western blot analysis of the sorted human ASCs; mouse brain serves as a positive control; (**D**) and (**E**) Representative photomicrographs of the cartilage-like tissues derived from HRST-*eGFP*-transduced ASCs (**D**) and HRST-*DCX-GP-eGFP*-transduced ASCs (**E**); (**F**) Western blot analysis of the proteins extracted from the cartilage-like tissues; (**G**) qRT-PCR analysis of gene expression in the cartilage-like tissues. Data represent mean ± SD (error bars) of three independent experiments; the difference between the ASC-*eGFP* and ASC-*DCX-GP-eGFP* groups was statistically significant (^*^ *p* < 0.05).

We constructed a lentiviral vector (HRST-*DCX-GP-eGFP*) to express human *DCX* in human adipose tissue-derived MSCs, also called adipose stem cells (ASCs). GP stands for glycine and proline within a consensus peptide sequence that automatically self-cleaves to separate DCX and eGFP proteins once *DCX-GP-eGFP* gene is translated based on a previous study [23]. As a control group, HRST-*eGFP* lentiviral vector was used. Human ASCs transduced with either HRST-*eGFP* or HRST-*DCX-GP-eGFP* lentiviruses were sorted out, based on eGFP expression (Figure 3A,B). DCX protein expression was confirmed by Western blot analysis (Figure 3C). Of note, DCX protein size was approximately 40 KDa, similar to the endogenous DCX protein expressed in mouse brain tissues, which indicates that the DCX-GP-eGFP fusion protein was indeed cleaved into separate DCX and eGFP proteins. DCX protein expression level was much lower in the transduced ASCs than the mouse brain tissues, which is comparable to the physiologic levels where DCX expression level in the limbs is dramatically less than in the brain and spinal cord [9]. Human ASCs with eGFP or DCX-GP-eGFP expression were cultured in pellets with chondrogenic media for 14 days. We found that both groups of human ASCs produced pieces of cartilage-like tissues with similar appearance (Figure 3D,E). Western blot analysis showed that DCX protein was expressed in the cartilage-like tissues derived from HRST-*DCX-GP-eGFP* lentivirus-transduced ASCs, but not in the cartilage-like tissues derived from HRST-*eGFP* lentivirus-transduced ASCs (Figure 3F). We checked a series of genes that are known to be expressed in articular or endochondral chondrocytes. We found that expression of collagen II was significantly decreased in the DCX-expressing pellets, whereas expression of aggrecan, matrilin 2, and *GDF5* was significantly increased in the DCX-expressing pellets (Figure 3G, *p* < 0.05). Superficial zone protein (*SZP*) was not detectable in either group. Collagen I is expressed by human ASCs. Expression of collagen I is expected to be reduced in chondrogenesis, however, we only observed a slight decrease in collagen I expression (Figure 3G). We speculate that this may be caused by an incomplete change from fibroblastic to chondrocytic phenotypes. It is paradoxical to observe that collagen II was reduced by DCX expression. However, we previously found that collagen II is expressed at higher levels in endochondral cartilage than articular cartilage [3], which suggests that less collagen II expression implies more articular chondrocytic phenotype than endochondral chondrocytic phenotype. Matrilin 2 and *GDF5* are restricted to articular chondrocytes [22–25]. DCX expression was decreased by 44-fold when E11.5 mouse limb bud mesenchymal cells were cultured in micromasses from day 3 to day 15 [23]. It is noteworthy that, in monolayer culture of mouse embryonic stem cells, GDF5 induced *DCX* expression on day four but its expression diminished over next eight days [26]. It is possible that *GDF5* and *DCX* provide reciprocal positive feedback in their expression, as both GDF5 and DCX proteins are restricted to articular cartilage. The differences in collagen II, matrilin 2, and *GDF5* between the two groups indicate that the DCX-expressing cartilage-like tissues lean towards expressing more genes that are specific for articular cartilage. Matrilin 1 levels were quite variable during our experiments (Figure 3G). However, since matrilin 1 is specific for endochondral chondrocytes [25], its increased expression in the DCX-expressing cartilage-like tissues argues against the speculation that *DCX* drives ASCs towards articular chondrocyte differentiation. Therefore, it awaits further investigation to clarify what *DCX*’s role is in chondrogenesis.

## 3. Experimental Section

### 3.1. Animals

Animal study was approved by the Institutional Animal Care and Use Committee of Tulane University (Protocol# 4040R, approved on 17 January 2011, valid through 16 January 2014). The *Dcx-EGFP* mice with a strain name of Tg (*Dcx-EGFP*) BJ224Gsat/Mmmh were obtained from the Mutant Mouse Regional Resource Center, University of Missouri, which were characterized previously [9]. Enhanced green fluorescence protein (eGFP) was expressed in *Dcx*-expressing cells in these mice. E12.5 and E13.5 mouse embryos were obtained through timed pregnancies. Images of mouse handplates were taken with an epifluorescence microscope (Nikon AZ100) equipped with a digital camera (Nikon DS-Qi1Mc) and NIS-Elements Basic 3.0 software (Nikon Instruments Inc., Melville, NY, USA). The handplates were dissected into different regions under the epifluorescence microscope, using VANNAS microdissecting spring scissors (Roboz Surgical, Gaithersburg, MD, USA). Approximately 16 handplates from 8 embryos of a single pregnant mouse each at E12.5 and E13.5 were collected and pooled.

### 3.2. RNA Extraction and Microarray

The dissected mouse embryonic tissues were homogenized and total RNA was extracted using RNeasy Mini Kit (QIAGEN, Valencia, CA, USA) with DNase I digestion to avoid genomic DNA contamination. RNA was dissolved in DNase/RNase-free water, quantified by a NanoDrop instrument (NanoDrop Products, part of Thermo Fisher Scientific, Wilmington, DE, USA) and set at a concentration of ~1.0 μg/μL. The quality of the RNA was confirmed by Agilent 2100 Bioanalyzer (Agilent Technologies, Palo Alto, CA, USA). Two hundred ng of RNA were used to make biotinylated cRNA using the Illumina TotalPrep RNA Amplification Kit (Ambion, Austin, TX, USA), and hybridized to the Illumina chips for 14 h at 58 °C. After washing and staining, the arrays were scanned with the BeadArray Reader (Illumina Inc., San Diego, CA, USA) and analyzed with the GenomeStudio software (Illumina Inc.) as described previously [27]. All microarray analysis was done at the LCRC Genomics Facility in New Orleans, LA, USA.

### 3.3. Microarray Data Analysis

After subtracting the background, the samples were normalized using the “cubic spline” algorithm assuming a similar distribution of transcript abundance in all the samples. Gene expression levels were compared to select only those genes with >2-fold differences (up or down-regulated) between the samples in comparison. All sequence data were assigned a gene ID corresponding to the Gene Symbol from the National Center for Biotechnology Information (NCBI) gene database [28]. These genes were then researched using both the NCBI database and the UniProt Protein Knowledgebase database [29] to annotate the corresponding protein function.

### 3.4. Cultures of Human Adipose Tissue-Derived Mesenchymal Stromal/Stem Cells

Human adipose tissue-derived mesenchymal stromal/stem cells (MSCs), also called adipose stem cells (ASCs), were collected at the Pennington Biomedical Research Center (Baton Rouge, LA, USA) with approval of the Institutional Review Board and all human participants provided written informed consent (PBRC #23040) as previously described [30,31]. The ASCs were provided to the researchers as de-identified materials. The ASCs were cultured in α-minimum essential medium (α-MEM, Mediatech Inc., Herndon, VA, USA) with 20% fetal bovine serum (FBS, Bio-West, Rosenberg, TX, USA) and 1% L-glutamine in a 37 °C, 5% CO_2_ humidified incubator.

### 3.5. Transduction of Human ASCs

HRST-eGFP lentiviral expression vector was derived from the original pHR’ CMV-lacZ vector [32], which expresses eGFP. Full-length human *DCX* cDNA was subcloned into HRST-*eGFP* vector through BamHI and XhoI sites, upstream to *eGFP*, thus, constructing HRST-*DCX-GP-eGFP* vector. GP stands for glycine and proline within a consensus peptide sequence that automatically self-cleaves to separate DCX and eGFP proteins once *DCX-GP-eGFP* gene was translated based on a previous study [33]. HRST-*eGFP* and HRST-*DCX-GP-eGFP* plasmids were individually packaged into replication-incompetent lentiviruses in 293T cells by co-transfection with packaging plasmids as described previously [34]. The packaging plasmids were pHDM-Hgpm2 (HIV gag-pol expression plasmid), pRC/CMV-Rev 1b (Accessory protein rev), pHDM-Tat 1b (Accessory protein tat), and pHDM.G (env, VSVG pseudotype). Human ASCs (within the first three passages following initial plating) were transduced with either HRST-eGFP or HRST-DCX-GP-eGFP lentiviruses for 16 h, and then thoroughly rinsed with phosphate-buffered saline (PBS) and cultured in complete medium. Forty-eight hours after transduction, the cells were harvested and eGFP-positive cells were sorted with a flow cytometry cell sorter (BD FACSAria, BD Biosciences, San Jose, CA, USA).

### 3.6. Pellet Culture

Approximately 200,000 eGFP+ or DCX-GP-eGFP+ ASCs were centrifuged in 15-mL conical polypropylene centrifuge tubes [13]. The cell pellets were cultured in chondrogenic media, that is, Dulbecco’s modified Eagle’s medium (DMEM) supplemented with 10 ng/mL BMP-7 (R&D Systems, Minneapolis, MN, USA), ITS solution (BD Biosciences, San Jose, CA, USA), 50 μg/mL 2-phospho-l-ascorbic acid trisodium salt (Sigma-Aldrich, St. Louis, MO, USA), 100 μg/mL sodium pyruvate (Invitrogen, Carlsbad, CA, USA), 100 nM dexamethasone (Sigma-Aldrich), 0.1% bovine serum albumin (Sigma-Aldrich). The medium was replaced every 3 days during 14 days of pellet culture.

### 3.7. Real-Time Quantitative Reverse Transcriptase PCR (qRT-PCR)

Pellets were homogenized and total RNA was extracted using RNeasy Mini Kit (QIAGEN, Valencia, CA, USA) with DNase I digestion to avoid genomic DNA contamination. cDNA was made from total RNA using iScript™ cDNA Synthesis Kit (Bio-Rad Laboratories, Hercules, CA, USA). PCR primers for human collagen I, collagen II, aggrecan, superficial zone protein (*SZP*), matrilin 1, matrilin 2, *GDF5*, and glyceraldehyde-3-phosphate dehydrogenase (*GAPDH*) were obtained from Eurofins MWG Operon (Huntsville, AL, USA) (Table 6). qRT-PCR was done in triplicates with an iQ5^®^ iCycler and iQ™ SYBR^®^ Green Supermix (Bio-Rad Laboratories, Hercules, CA, USA) following the recommended protocols. Results were normalized to *GAPDH* levels using the formula Δ*C*_t_ (Cycle threshold) = *C*_t_ of target gene − *C*_t_ of *GAPDH*. The mRNA level of the ASCs transduced with HRST-eGFP lentiviruses was used as the baseline; therefore, ΔΔ*C*_t_ was calculated using the formula ΔΔ*C*_t_ = Δ*C*_t_ of the target gene − Δ*C*_t_ of the baseline. The fold change of mRNA level was calculated as fold = 2^−ΔΔ^*^C^*^t^ [35]. Three independent experiments were conducted and data represent mean ± SD (error bars) of 3 independent experiments.

### 3.8. Western Blot Analysis

Human ASCs (after flow cytometry sorting) and homogenates of the ASCs pellets were lysed with lysis buffer (50 mM sodium fluoride, 0.5% Igepal CA-630 (NP-40), 10 mM sodium phosphate, 150 mM sodium chloride, 25 mM Tris pH 8.0, 1 mM phenylmethylsulfonyl fluoride, 2 mM ethylenediaminetetraacetic acid (EDTA), 1.2 mM sodium vanadate) supplemented with protease inhibitor cocktail (Sigma-Aldrich, St. Louis, MO, USA). Equal amount of proteins was subjected to 10% SDS-polyacrylamide gel electrophoresis and transferred to polyvinylidene difluoride membrane. Protein extract from mouse brain tissues was used as a positive control for DCX protein [36]. The membranes were blocked with 5% nonfat dry milk in TBST buffer (25 mM Tris-HCl, 125 mM NaCl, 0.1% Tween 20) for 2 h and incubated with goat anti-DCX antibodies (sc-8066, Santa Cruz Biotechnology, Santa Cruz, CA, USA) overnight and then IRDye^®^800CW-conjugated donkey anti-goat secondary antibodies (LI-COR Biosciences, Lincoln, NE, USA) for 1 h. The results were visualized by using an Odyssey Infrared Imager (LI-COR Biosciences, Lincoln, NE, USA). For loading control, the membranes were also probed for GAPDH using mouse anti-GAPDH antibodies (MAB374, Millipore Corporation, Billerica, MA, USA).

### 3.9. Statistical Analysis

Student’s *t*-test (two-tailed) was used to analyze the qRT-PCR data and *p*-value <0.05 was considered statistically significant.

## 4. Conclusions

The present study used *DCX* promoter-driven *eGFP* expression as a guide to dissect different regions of E12.5 and E13.5 mouse embryonic handplates. Microarray analysis of gene expression profiles identified a variety of genes that were expressed differentially in the different regions of mouse handplate *in vivo*. The unique expression patterns of several genes, e.g., *Cytl1*, are intriguing targets for further investigation. The *in vitro* experiments showed that DCX affected expression of several genes associated with chondrocyte phenotype, such as collagen II, aggrecan, matrilin 2, and *GDF5*. These findings imply that *DCX* may play a role in driving differentiation of articular chondrocyte phenotype, which awaits future studies for further clarification.

## Supplementary Information



## Figures and Tables

**Table 1.** Differential gene expression between the proximal and distal regions of E12.5 mouse handplate.

No.	Gene ID	Gene Symbol	Proximal/Distal Ratio of Expression	Gene Name	Function
1	13386	*Dlk1*	14.40	*Delta-like 1 homolog (Drosophila)*	Inhibitory non-canonical protein ligand for the NOTCH1 receptor
2	15464	*Hrc*	4.84	*Histidine rich calcium binding protein*	Interactions with SERCA2 and triadin
3	231162	*Cytl1*	4.53	*Cytokine-like 1*	Cytl1-null mice show normal cartilage and bone development but exhibit augmented osteoarthritic cartilage destruction
4	53412	*Ppp1r3c*	3.19	*Protein phosphatase 1, regulatory (inhibitor) subunit 3C*	Activates glycogen synthase, reduces glycogen phosphorylase activity and limits glycogen breakdown
5	16664	*Krt14*	3.15	*Keratin 14*	Enhances the mechanical properties involved in resilience of keratin intermediate filaments
6	74194	*Rnd3*	2.90	*Rho family GTPase 3*	Binds GTP but lacks intrinsic GTPase activity
7	12306	*Anxa2*	2.39	*Annexin A2*	Calcium-regulated membrane-binding protein
8	14314	*Fstl*	2.34	*Follistatin-like 1*	May modulate the action of some growth factors on cell proliferation and differentiation
9	21847	*Klf10*	2.32	*Kruppel-like factor 10*	Inhibits cell growth
10	16002	*Igf2*	2.31	*Insulin-like growth factor 2*	Growth-promoting activity
11	17709	*Mt-co2*	2.18	*Cytochrome c oxidase subunit II*	Component of the respiratory chain
12	73121	*3110032G18RIK*	2.05	*Family with sequence similarity 101, member A*	Unknown
13	15401	*Hoxa4*	0.49	*Homeobox A4*	Sequence-specific transcription factor
14	15117	*Has2*	0.48	*Hyaluronan synthase 2*	Hyaluronan/hyaluronic acid (HA) synthesis
15	77087	*3010027A04RIK*	0.44	*Ankyrin repeat domain 11*	Bone development
16	258201	*Olfr538*	0.43	*Olfactory receptor 538*	Interact with odorant molecules in the nose
17	233107	*Kctd15*	0.39	*Potassium channel tetramerisation domain containing 15*	Unknown
18	12705	*Cited1*	0.36	*Cbp/p300-interacting transactivator with Glu/Asp-rich carboxy-terminal domain 1*	Transcriptional coactivator of the p300/CBP-mediated trancription complex
19	20204	*Prrx2*	0.29	*Paired related homeobox 2*	A developmental protein

**Table 2.** Differential gene expression between the carpal and metacarpal regions of E13.5 mouse handplate.

No.	Gene ID	Gene Name	Carpal/Metacarpal Ratio of Expression	Gene Name	Function
1	73121	*3110032G18rik*	4.55	*Family with sequence similarity 101*, *member A*	A novel gene uniquely expressed in developing forebrain and midbrain, but its null mutant exhibits no obvious phenotype
2	231162	*Cytl1*	3.54	*Cytokine-like 1*	Cytl1-null mice show normal cartilage and bone development but exhibit augmented osteoarthritic cartilage destruction
3	50781	*Dkk3*	3.26	*Dickkopf homolog 3 (Xenopus laevis)*	Antagonizes canonical Wnt signaling
4	77853	*Msl2*	2.17	*Male-specific lethal 2 homolog (Drosophila)*	Promotes Mdm2-independent cytoplasmic localization of p53
5	17709	*Mt-co2*	0.46	*Cytochrome c oxidase subunit II*	Component of the respiratory chain
6	21804	*Tgfb1I1*	0.46	*Transforming growth factor beta 1 induced transcript 1*	A molecular adapter coordinating multiple protein-protein interactions
7	233107	*Kctd15*	0.46	*Potassium channel tetramerisation domain containing 15*	Unknown
8	93897	*Fzd10*	0.45	*Frizzled homolog 10 (Drosophila)*	Receptor for Wnt proteins
9	258201	*Olfr538*	0.45	*Olfactory receptor 538*	Interact with odorant molecules in the nose
10	106565	*Dlk2*	0.42	*Delta-like 2 homolog (Drosophila)*	Acts as inhibitory non-canonical protein ligands for the NOTCH1 receptor
11	12705	*Cited1*	0.41	*Cbp/p300-interacting transactivator with Glu/Asp-rich carboxy-terminal domain 1*	Transcriptional coactivator of the p300/CBP-mediated trancription complex
12	15464	*Hrc*	0.32	*Histidine rich calcium binding protein*	May play a key role in the regulation of SR Ca cycling through its direct interactions with SERCA2 and triadin
13	54419	*Cldn6*	0.29	*Claudin 6*	Plays a major role in tight junction-specific obliteration of the intercellular space, through calcium-independent cell-adhesion
14	16664	*Krt14*	0.15	*keratin 14*	The nonhelical tail domain is involved in promoting KRT5-KRT14 filaments to self-organize into large bundles

**Table 3.** Differential gene expression between the metacarpal and metacarpal-phalange regions of E13.5 mouse handplate.

No.	Gene ID	Gene Symbol	Metacarpal/Metacarpal-Phalange Ratio of Expression	Gene Name	Function
1	13386	*Dlk1*	2.64	*Delta-like 1 homolog (Drosophila)*	Acts as inhibitory non-canonical protein ligand for the NOTCH1 receptor
2	71706	*Slc46a3*	2.35	*Solute carrier family 46*, *member 3*	Unknown
3	11806	*Apoa1*	2.25	*Apolipoprotein A-I*	Reverse transport of cholesterol from tissues to the liver for excretion
4	54419	*Cldn6*	2.15	*Claudin 6*	Role in tight junction-specific obliteration of the intercellular space, through calcium-independent cell-adhesion activity
5	21804	*Tgfb1I1*	2.12	*Transforming growth factor beta 1 induced transcript 1*	Functions as a molecular adapter coordinating multiple protein-protein interactions at the focal adhesion complex in nucleus
6	12709	*Ckb*	2.06	*Creatine kinase*, *brain*	Phospholipid biosynthesis
7	11472	*Actn2*	0.48	*Actinin alpha 2*	F-actin cross-linking protein which is thought to anchor actin to a variety of intracellular structures
8	15464	*Hrc*	0.48	*Histidine rich calcium binding protein*	Regulation of SR Ca cycling through its direct interactions with SERCA2 and triadin
9	16876	*Lhx9*	0.46	*LIM homeobox protein 9*	Gonadal development
10	56360	*Acot9*	0.46	*Acyl-CoA thioesterase 9*	Catalyze the hydrolysis of acyl-CoAs to the free fatty acid and coenzyme A
11	72739	*Zkscan3*	0.46	*Zinc finger with KRAB and SCAN domains 3*	Acts as a transcriptional regulator
12	16704	*Krtap8-2*	0.45	*Keratin associated protein 8-2*	Essential for the formation of a rigid and resistant hair shaft through their extensive disulfide bond cross-linking
13	68895	*Rasl11a*	0.44	*RAS-like*, *family 11*, *member A*	Regulator of rDNA transcription. Acts in cooperation UBF/UBTF and positively regulates RNA polymerase I transcription
14	17883	*Myh3*	0.39	*Myosin*, *heavy polypeptide 3*, *skeletal muscle*, *embryonic*	Mutations in this gene have been associated Freeman-Sheldon syndrome and Sheldon-Hall syndrome
15	17885	*Myh8*	0.38	*Myosin*, *heavy polypeptide 8*, *skeletal muscle*, *perinatal*	Motor protein of muscle thick filaments
16	19791	*Rn18s*	0.28	*18S Ribosomal RNA*	A 45S rRNA, which serves as the precursor for the 18S, 5.8S and 28S rRNA, is transcribed from rDNA unit by RNA polymerase I
17	226856	*Lpgat1*	0.26	*Lysophosphatidylglycerol acyltransferase 1*	Recognizes various acyl-CoAs and LPGs as substrates but demonstrates a clear preference

**Table 4.** Differential gene expression between the proximal region of E12.5 handplate and the carpal region of E13.5 mouse handplate.

No.	Gene ID	Gene Symbol	E12.5 Proximal/E13.5 Carpal Ratio of Expression	Gene Name	Function
1	231162	*Cytl1*	18.15	*Cytokine-like 1*	Cytl1-null mice show normal cartilage and bone development but exhibit augmented osteoarthritic cartilage destruction.
2	73121	*3110032G18rik*	7.09	*Family with sequence similarity 101*, *member A*	A novel gene uniquely expressed in developing forebrain and midbrain
3	50781	*Dkk3*	6.15	*Dickkopf homolog 3 (Xenopus laevis)*	Antagonizes canonical Wnt signaling
4	19791	*Rn18s*	6.07	*18S ribosomal RNA*	Encodes a 18S rRNA
5	319480	*Itga11*	3.89	*Integrin alpha 11*	Regulating Bone morphogenetic protein (BMP)-2 and transforming growth factor (TGF)-beta1
6	13386	*Dlk1*	2.93	*Delta-like 1 homolog (Drosophila)*	Acts as inhibitory non-canonical protein ligand for the NOTCH1 receptor
7	15401	*Hoxa4*	2.70	*Homeobox A4*	Sequence-specific transcription factor
8	20680	*Sox7*	2.29	*SRY-box containing gene 7*	A member of the SOX (SRY-related HMG-box) family of transcription factors involved in regulation
9	67586	*D4bwg1540e*	2.29	*UBX domain protein 11*	May be involved in the reorganization of actin cytoskeleton mediated by RND1, RND2, and RND3
10	26433	*Plod3*	2.11	*Procollagen-lysine*, *2-oxoglutarate 5-dioxygenase 3*	Forms hydroxylysine residues in -Xaa-Lys-Gly- sequences in collagens
11	100034361	*Mfap1b*	0.48	*Microfibrillarassociated protein 1B*	Component of the elastin-associated microfibrils By similarity
12	21371	*Tbca*	0.47	*Tubulin cofactor A*	Tubulin-folding protein; involved in the early step of the tubulin folding pathway
13	26941	*Slc9a3r1*	0.41	*Solute carrier family 9 (sodium/hydrogen exchanger), member 3 regulator 1*	Scaffold protein that connects plasma membrane proteins with members of the ezrin/moesin/radixin
14	12301	*Cacybp*	0.29	*Calcyclin binding protein*	CacyBP/SIP interacts with tubulin in neuroblastoma NB2a cells and induces formation of globular tubulin assemblies.
15	93897	*Fzd10*	0.25	*Frizzled homolog 10 (Drosophila)*	Receptor for Wnt proteins. Most of frizzled receptors are coupled to the beta-catenin canonical signaling pathway
16	18590	*Pdgfa*	0.24	*Platelet derived growth factor, alpha*	Growth factor that plays an essential role in the regulation of embryonic development
17	66643	*Lix1*	0.24	*Limb expression 1 homolog (chicken)*	Unknown
18	16664	*Krt14*	0.15	*Keratin 14*	Involved in resilience of keratin intermediate filaments

**Table 5.** Differential gene expression between the proximal region of E12.5 handplate and the metacarpal-phalange region of E13.5 mouse handplate.

No.	Gene ID	Gene Symbol	E12.5 Proximal/E13.5 Metacarpal-Phalange Ratio of Expression	Gene Name	Function
1	19791	*Rn18s*	6.66	*Rn18s 18S ribosomal RNA*	Encodes a 18S rRNA
2	319480	*Itga11*	4.54	*Integrin alpha 11*	Regulating Bone morphogenetic protein (BMP)-2 and transforming growth factor (TGF)-beta1
3	231162	*Cytl1*	4.09	*Cytokine-like 1*	Cytl1-null mice show normal cartilage and bone development but exhibit augmented osteoarthritic cartilage destruction.
4	72053	*2010008E23Rik*	3.93	*Transmembrane and ubiquitin-like domain containing 2*	Unknown
5	21804	*Tgfb1i1*	3.16	*Transforming growth factor beta 1 induced transcript 1*	A molecular adapter coordinating multiple protein-protein interactions at the focal adhesion complex and in the nucleus
6	108903	*Tbcd*	2.79	*Tubulin-specific chaperone d*	Tubulin-folding protein
7	67586	*Ubxn11*	2.3	*UBX domain protein 11*	Reorganization of actin cytoskeleton
8	20680	*Sox7*	2.24	*SRY-box containing gene 7*	member of the SOX (SRY-related HMG-box) family of transcription factors
9	50781	*Dkk3*	2.16	*Dickkopf homolog 3 (Xenopus laevis)*	Inhibit Wnt regulated processes
10	258201	*Olfr538*	2.06	*Olfactory receptor 538*	Olfactory receptors interact with odorant molecules in the nose
11	100034361	*Mfap1b*	0.45	*Microfibrillar-associated protein 1B*	Component of the elastin-associated microfibrils by similarity
12	66643	*Lix1*	0.15	*Limb expression 1 homolog (chicken)*	Little is known about LIX1, except that it is evolutionarily conserved and highly expressed in spinal cord motor neurons

**Table 6.** Nucleotide sequences of each PCR primer pair.

Gene	Primer	Nucleotide Sequence (5′ to 3′)
*Collagen I*	sense	CACCAATCACCTGCGTACAGAA
	antisense	ACAGATCACGTCATCGCACAAC

*Collagen II*	sense	GGCAATAGCAGGTTCACGTACA
	antisense	CGATAACAGTCTTGCCCCACTT

*Aggrecan (core protein)*	sense	AAGTATCATCAGTCCCAGAATCTAGCA
	antisense	CGTGGAATGCAGAGGTGGTT

*SZP*	sense	TTGCGCAATGGGACATTAGTT
	antisense	AGCTGGAGATGGTGGACTGAA

*Matrilin 1*	sense	AGGGACTGCGTTTGCATTTTT
	antisense	TCAGTAAAGAAATTCACAGCACTCAGA

*Matrilin 2*	sense	GACGGACGGGCTCAGGAT
	antisense	GATACCATTGGCCTTGGCTTTA

*GDF5*	sense	ATTTGTGCCTGGTGACTTCC
	antisense	AGCCCTCTCCTCTTCTCTCC

*GAPDH*	sense	TAAAAGCAGCCCTGGTGACC
	antisense	CCACATCGCTCAGACACCAT

## References

[1] Singh G., Miller J.D., Lee F.H., Pettitt D., Russell M.W. (2002). Prevalence of cardiovascular disease risk factors among US adults with self-reported osteoarthritis: Data from the third national health and nutrition examination survey. Am. J. Manag. Care.

[2] Tuan R.S. (2006). Stemming cartilage degeneration: Adult mesenchymal stem cells as a cell source for articular cartilage tissue engineering. Arthritis Rheumatol.

[3] Yamane S., Cheng E., You Z., Reddi A.H. (2007). Gene expression profiling of mouse articular and growth plate cartilage. Tissue Eng.

[4] Muneoka K., Bryant S.V. (1982). Evidence that patterning mechanisms in developing and regenerating limbs are the same. Nature.

[5] Reddi A.H. (1998). Cartilage-derived morphogenetic proteins and cartilage morphogenesis. Microsc. Res. Tech.

[6] Martin P. (1990). Tissue patterning in the developing mouse limb. Int. J. Dev. Biol.

[7] Mitrovic D. (1978). Development of the diarthrodial joints in the rat embryo. Am. J. Anat.

[8] Koyama E., Shibukawa Y., Nagayama M., Sugito H., Young B., Yuasa T., Okabe T., Ochiai T., Kamiya N., Rountree R.B. (2008). A distinct cohort of progenitor cells participates in synovial joint and articular cartilage formation during mouse limb skeletogenesis. Dev. Biol.

[9] Zhang Q., Cigan A.D., Marrero L., Lopreore C., Liu S., Ge D., Savoie F.H., You Z. (2011). Expression of doublecortin reveals articular chondrocyte lineage in mouse embryonic limbs. Genesis.

[10] Bai J., Ramos R.L., Ackman J.B., Thomas A.M., Lee R.V., LoTurco J.J. (2003). RNAi reveals doublecortin is required for radial migration in rat neocortex. Nat. Neurosci.

[11] Gleeson J.G., Allen K.M., Fox J.W., Lamperti E.D., Berkovic S., Scheffer I., Cooper E.C., Dobyns W.B., Minnerath S.R., Ross M.E. (1998). Doublecortin, a brain-specific gene mutated in human X-linked lissencephaly and double cortex syndrome, encodes a putative signaling protein. Cell.

[12] Des Portes V., Francis F., Pinard J.M., Desguerre I., Moutard M.L., Snoeck I., Meiners L.C., Capron F., Cusmai R., Ricci S. (1998). Doublecortin is the major gene causing X-linked subcortical laminar heterotopia (SCLH). Hum. Mol. Genet.

[13] Zhang Y., Ryan J.A., di Cesare P.E., Liu J., Walsh C.A., You Z. (2007). Doublecortin is expressed in articular chondrocytes. Biochem. Biophys. Res. Commun.

[14] Chen L., Qanie D., Jafari A., Taipaleenmaki H., Jensen C.H., Saamanen A.M., Sanz M.L., Laborda J., Abdallah B.M., Kassem M. (2011). Delta-like 1/fetal antigen-1 (Dlk1/FA1) is a novel regulator of chondrogenic cell differentiation via inhibition of the Akt kinase-dependent pathway. J. Biol. Chem.

[15] Kim J.S., Ryoo Z.Y., Chun J.S. (2007). Cytokine-like 1 (*Cytl1*) regulates the chondrogenesis of mesenchymal cells. J. Biol. Chem.

[16] Jeon J., Oh H., Lee G., Ryu J.H., Rhee J., Kim J.H., Chung K.H., Song W.K., Chun C.H., Chun J.S. (2011). Cytokine-like 1 knock-out mice (*Cytl1*−/−) show normal cartilage and bone development but exhibit augmented osteoarthritic cartilage destruction. J. Biol. Chem.

[17] Leussink B., Brouwer A., el Khattabi M., Poelmann R.E., Gittenberger-de Groot A.C., Meijlink F. (1995). Expression patterns of the paired-related homeobox genes MHox/Prx1 and S8/Prx2 suggest roles in development of the heart and the forebrain. Mech. Dev.

[18] Minogue B.M., Richardson S.M., Zeef L.A., Freemont A.J., Hoyland J.A. (2010). Characterization of the human nucleus pulposus cell phenotype and evaluation of novel marker gene expression to define adult stem cell differentiation. Arthritis Rheumatol.

[19] Liu S., Zhang E., Yang M., Lu L. (2014). Overexpression of Wnt11 promotes chondrogenic differentiation of bone marrow-derived mesenchymal stem cells in synergism with TGF-β. Mol. Cell. Biochem.

[20] Zanotti S., Canalis E. (2013). Notch suppresses nuclear factor of activated T cells (NFAT) transactivation and Nfatc1 expression in chondrocytes. Endocrinology.

[21] Yamagami T., Molotkov A., Zhou C.J. (2009). Canonical Wnt signaling activity during synovial joint development. J. Mol. Histol.

[22] Pitsillides A.A., Beier F. (2011). Cartilage biology in osteoarthritis—Lessons from developmental biology. Nat. Rev. Rheumatol.

[23] James C.G., Appleton C.T., Ulici V., Underhill T.M., Beier F. (2005). Microarray analyses of gene expression during chondrocyte differentiation identifies novel regulators of hypertrophy. Mol. Biol. Cell.

[24] Klatt A.R., Paulsson M., Wagener R. (2002). Expression of matrilins during maturation of mouse skeletal tissues. Matrix Biol.

[25] Segat D., Frie C., Nitsche P.D., Klatt A.R., Piecha D., Korpos E., Deak F., Wagener R., Paulsson M., Smyth N. (2000). Expression of matrilin-1, -2 and -3 in developing mouse limbs and heart. Matrix Biol.

[26] Craft A.M., Ahmed N., Rockel J.S., Baht G.S., Alman B.A., Kandel R.A., Grigoriadis A.E., Keller G.M. (2013). Specification of chondrocytes and cartilage tissues from embryonic stem cells. Development.

[27] Kim S.H., Sierra R.A., McGee D.J., Zabaleta J. (2012). Transcriptional profiling of gastric epithelial cells infected with wild type or arginase-deficient Helicobacter pylori. BMC Microbiol.

[28] National Center for Biotechnology Information (NCBI) Gene Database http://www.ncbi.nlm.nih.gov/.

[29] UniProt Protein Knowledgebase Database http://www.uniprot.org/.

[30] Yu G., Wu X., Dietrich M.A., Polk P., Scott L.K., Ptitsyn A.A., Gimble J.M. (2010). Yield and characterization of subcutaneous human adipose-derived stem cells by flow cytometric and adipogenic mRNA analyzes. Cytotherapy.

[31] Strong A.L., Strong T.A., Rhodes L.V., Semon J.A., Zhang X., Shi Z., Zhang S., Gimble J.M., Burow M.E., Bunnell B.A. (2013). Obesity associated alterations in the biology of adipose stem cells mediate enhanced tumorigenesis by estrogen dependent pathways. Breast Cancer Res.

[32] Naldini L., Blomer U., Gallay P., Ory D., Mulligan R., Gage F.H., Verma I.M., Trono D. (1996). *In vivo* gene delivery and stable transduction of nondividing cells by a lentiviral vector. Science.

[33] Szymczak A.L., Workman C.J., Wang Y., Vignali K.M., Dilioglou S., Vanin E.F., Vignali D.A. (2004). Correction of multi-gene deficiency *in vivo* using a single ‘self-cleaving’ 2A peptide-based retroviral vector. Nat. Biotechnol.

[34] Mostoslavsky G., Kotton D.N., Fabian A.J., Gray J.T., Lee J.S., Mulligan R.C. (2005). Efficiency of transduction of highly purified murine hematopoietic stem cells by lentiviral and oncoretroviral vectors under conditions of minimal *in vitro* manipulation. Mol. Ther.

[35] Ge D., Dauchy R.T., Liu S., Zhang Q., Mao L., Dauchy E.M., Blask D.E., Hill S.M., Rowan B.G., Brainard G.C. (2013). Insulin and IGF1 enhance IL-17-induced chemokine expression through a GSK3B-dependent mechanism: a new target for melatonin’s anti-inflammatory action. J. Pineal Res.

[36] Corbo J.C., Deuel T.A., Long J.M., LaPorte P., Tsai E., Wynshaw-Boris A., Walsh C.A. (2002). Doublecortin is required in mice for lamination of the hippocampus but not the neocortex. J. Neurosci.

