# Supplementary material for: Doublecortin May Play a Role in Defining Chondrocyte Phenotype
Source: Int J Mol Sci. 2014 Apr 22;15(4):6941–60. doi: 10.3390/ijms15046941 (PMC4013671; doi:10.3390/ijms15046941)
Supplement: Supplementary file 1 [file ijms-15-06941-s001.pdf]

Supplementary Information

Table S1. Differential gene expression between the proximal and distal regions of E12.5 mouse handplate.

No.	Gene ID	Gene Symbol	Proximal/Distal Ratio of Expression	Gene Name	Function
1	17883	*Myh3*	69.53	*Myosin, heavy polypeptide 3, skeletal muscle, embryonic*	Associated with arthrogryposis syndromes, Freeman-Sheldon syndrome and Sheldon-Hall syndrome
2	21955	*Tnnt1*	33.21	*Troponin T1, skeletal, slow*	Tropomyosin-binding subunit
3	21924	*Tnnc1*	31.61	*Troponin C, cardiac/slow skeletal*	Central regulatory protein of striated muscle contraction
4	17896	*Myl4*	23.24	*Myosin, light polypeptide 4*	Regulatory light chain of myosin
5	11472	*Actn2*	14.71	*Actinin alpha 2*	F-actin cross-linking protein
6	17885	*Myh8*	12.62	*Myosin, heavy polypeptide 8, skeletal muscle, perinatal*	Motor protein of muscle thick filaments
7	16826	*Ldb2*	3.98	*LIM domain binding 2*	Binds to the LIM domain of a wide variety of LIM domain-containing transcription factors
8	231440	*9130213B05RIK*	3.77	*Prostate androgen-regulated mucin-like protein 1*	May regulate TLP1 expression and telomerase activity, thus enabling certain prostatic cells to resist apoptosis
9	140709	*Emid2*	2.68	*EMI domain containing 2*	Unknown
10	328232	*Gfod1*	2.66	*Glucose-fructose oxidoreductase domain containing 1*	Unknown
11	14955	*H19*	2.65	*H19 fetal liver mRNA*	Activated in extraembryonic cell at implantation
12	668171	*Zxda*	2.60	*Zinc finger, X-linked, duplicated A*	Cooperates with CIITA to promote transcription of MHC class I and MHC class II genes
13	66643	*Lix1*	2.47	*Limb expression 1 homolog (chicken)*	Evolutionarily conserved and highly expressed in spinal cord motor neurons
14	12709	*Ckb*	2.35	*Creatine kinase, brain*	Phospholipid biosynthesis
15	66214	*1190002H23RIK*	2.31	*RIKEN cDNA 1190002H23 gene*	Enhances CDK1 activity
16	67026	*Thap4*	2.20	*THAP domain containing 4*	Unknown
17	319747	*C130023A14RIK*	2.15	*RIKEN cDNA C130023A14 gene*	Unknown
18	100040413	*LOC100040413*	2.14	*Predicted gene 10075*	Unknown
19	66943	*Pqlc1*	2.06	*PQ loop repeat containing 1*	Unknown

Table S1. *Cont.*

No.	Gene ID	Gene Symbol	Proximal/Distal Ratio of Expression	Gene Name	Function
20	83922	*Tsga14*	2.06	*Testis specific gene A14*	Unknown
21	208213	*TMEM132C*	2.06	*transmembrane protein 132C*	Negative regulation of phosphatase activity
22	74718	*Snx16*	2.00	*sorting nexin 16*	Protein intracellular trafficking
23	16898	*Rps2*	0.49	*Ribosomal protein S2*	Unknown
24	666676	*LOC666676*	0.49	*Predicted gene 8230*	Unknown
25	66409	*Rsl1d1*	0.48	*Ribosomal L1 domain containing 1*	Unknown
26	72053	*2010008E23RIK*	0.48	*Transmembrane and ubiquitin-like domain containing 2*	Unknown
27	225849	*Ppp2r5b*	0.48	*Protein phosphatase 2, regulatory subunit B (B56), beta isoform*	Unknown
28	56457	*Clptm1*	0.47	*Cleft lip and palate associated transmembrane protein 1*	T-cell development
29	217365	*Nploc4*	0.47	*Nuclear protein localization 4 homolog (S. cerevisiae)*	Binds ubiquitinated proteins
30	224897	*Dpp9*	0.47	*Dipeptidylpeptidase 9*	Peptidase
31	258293	*Olfr437*	0.47	*olfactory receptor 437*	Interact with odorant molecules in the nose
32	22174	*Tyro3*	0.46	*TYRO3 protein tyrosine kinase 3*	Receptor kinase that transduces signals from the extracellular matrix into the cytoplasm by binding several ligands
33	66525	*Timm50*	0.46	*Translocase of inner mitochondrial membrane 50 homolog (yeast)*	Essential component of the TIM23 complex
34	319562	*9630028B13RIK*	0.46	*RIKEN cDNA 9630028B13 gene*	Unknown
35	26462	*Txnrd2*	0.45	*Thioredoxin reductase 2*	Maintains thioredoxin in a reduced state
36	13361	*Dhfr*	0.44	*Dihydrofolate reductase*	Folate metabolism
37	52846	*D1bwg0212e*	0.44	*DNA segment, Chr 1, Brigham & Women's Genetics 0212 expressed*	Unknown

Table S1. *Cont.*

No.	Gene ID	Gene Symbol	Proximal/Distal Ratio of Expression	Gene Name	Function
38	272396	*Tarsl2*	0.44	*Threonyl-tRNA synthetase-like 2*	Unknown
39	434204	*Whdc1*	0.44	*WAS protein homolog associated with actin, golgi membranes and microtubules*	Stimulates Arp2/3-mediated actin polymerization at the Golgi apparatus and along tubular membranes
40	68544	*2310036O22RIK*	0.43	*RIKEN cDNA 2310036O22 gene*	Unknown
41	69168	*Bola1*	0.43	*bolA-like 1 (E. coli)*	Unknown
42	319581	*Xkr5*	0.42	*X Kell blood group precursor-related family, member 5*	Unknown Function
43	68895	*Rasl11a*	0.41	*RAS-like, family 11, member A*	Regulator of rDNA transcription
44	66570	*Cenpm*	0.39	*Centromere protein M*	Assembly of kinetochore proteins
45	319480	*Itga11*	0.39	*Integrin alpha 11*	Regulating Bone morphogenetic protein (BMP)-2 and transforming growth factor (TGF)-beta1
46	69719	*Cad*	0.38	*Carbamoyl-phosphate synthetase 2, aspartate transcarbamylase, and dihydroorotase*	Unknown Function
47	94065	*Mrpl34*	0.38	*Mitochondrial ribosomal protein L34*	Unknown
48	68195	*Rnaset2*	0.37	*Ribonuclease T2B*	Unknown
49	70020	*Znhit4*	0.37	*INO80 complex subunit B*	Core component of the INO80 complex
50	54632	*Ftsj1*	0.36	*FtsJ homolog 1 (E. coli)*	Unknown Function
51	235504	*Slc17a5*	0.35	*Solute carrier family 17 (anion/sugar transporter), member 5*	Primary solute translocator for anionic substances
52	16876	*Lhx9*	0.34	*LIM homeobox protein 9*	Gonadal development.
53	20393	*Sgk1*	0.34	*Serum/glucocorticoid regulated kinase 1*	Regulation of a wide variety of ion channels
54	16783	*Lamp1*	0.33	*lysosomal-associated membrane protein 1*	Presents carbohydrate ligands to selectins

Table S1. *Cont.*

No.	Gene ID	Gene Symbol	Proximal/Distal Ratio of Expression	Gene Name	Function
55	107951	*Cdk9*	0.32	*Cyclin-dependent kinase 9 (CDC2-related kinase)*	Regulation of transcription
56	19791	*Rn18s*	0.31	*18S ribosomal RNA*	Encodes a 18S rRNA
57	192191	*Med9*	0.30	*Mediator of RNA polymerase II transcription, subunit 9 homolog (yeast)*	Regulates transcription of nearly all RNA polymerase II-dependent genes
58	621893	*Hist2h2ab*	0.28	*Histone cluster 2, H2ab*	Core component of nucleosome
59	11806	*Apoa1*	0.10	*apolipoprotein A-I*	Reverse transport of cholesterol from tissues to the liver for excretion

Table S2. Differential gene expression between the carpal and metacarpal regions of E13.5 mouse handplate.

No.	Gene ID	Gene Name	Carpal/Metacarpal Ratio of Expression	Gene Name	Function
1	15214	*Hey2*	0.49	*Hairy/enhancer-of-split related with YRPW motif 2*	Transcriptional repressor
2	15528	*Hspe1*	0.49	*Heat shock protein 1 (chaperonin 10)*	Inhibits lipopolysaccharide-induced inflammatory mediator production
3	103172	*Ndg2*	0.49	*Coiled-coil-helix-coiled-coil-helix domain containing 10*	Unknown
4	12301	*Ccybp*	0.47	*Calcyclin binding protein*	CacyBP/SIP interacts with tubulin induces formation of globular tubulin assemblies
5	22437	*Cmya1*	0.45	*Xin actin-binding repeat containing 1*	Xin is an actin binding protein which protects actin filaments from depolymerization and is expressed within muscle satellite cells
6	26950	*Vsnl1*	0.45	*Visinin-like 1*	Regulates the inhibition of rhodopsin phosphorylation in a calcium-dependent manner
7	170812	*Eraf*	0.45	*Alpha hemoglobin stabilizing protein*	Chaperone for free alpha-hemoglobin (alpha-Hb)
8	67712	*Slc25a37*	0.44	*Solute carrier family 25, member 37*	Iron transporter
9	17748	*Mt1*	0.42	*Metallothionein 1*	These proteins are transcriptionally regulated by both heavy metals and glucocorticoids
10	17896	*Myl4*	0.42	*Myosin, light polypeptide 4*	Regulatory light chain of myosin
11	17883	*Myh3*	0.41	*Myosin, heavy polypeptide 3, skeletal muscle, embryonic*	Mutations in this gene have been associated with Freeman-Sheldon syndrome and Sheldon-Hall syndrome

Table S2. *Cont.*

No.	Gene ID	Gene Name	Carpal/Metacarpal Ratio of Expression	Gene Name	Function
12	17885	*Myh8*	0.41	*Myosin, heavy polypeptide 8, skeletal muscle, perinatal*	Motor protein of muscle thick filaments
13	21380	*Tbx1*	0.39	*T-box 1*	Important regulatory gene in development, highly expressed in hair follicle (HF) stem cells
14	21955	*Tnnt1*	0.37	*Troponin T1, skeletal, slow*	The tropomyosin-binding subunit of troponin, the thin filament regulatory complex which confers calcium-sensitivity
15	68895	*Rasl11a*	0.37	*RAS-like, family 11, member A*	Regulator of rDNA transcription. Acts in cooperation UBF/UBTF and positively regulates RNA polymerase I transcription
16	11472	*Actn2*	0.34	*Actinin alpha 2*	F-actin cross-linking protein which is thought to anchor actin to a variety of intracellular structures
17	109978	*Art4*	0.3	*ADP-ribosyltransferase 4*	may be part of a novel protein family with an important role in regulating cell function
18	21924	*Tnnc1*	0.25	*Troponin C, cardiac/slow skeletal*	Troponin is the central regulatory protein of striated muscle contraction.

Table S3. Differential gene expression between the proximal region of E12.5 handplate and the carpal region of E13.5 mouse handplate.

No.	Gene ID	Gene Symbol	E12.5 Proximal/E13.5 Carpal Ratio of Expression	Gene Name	Function
1	17885	*Myh8*	3.65	*Myosin, heavy polypeptide 8, skeletal muscle, perinatal*	Motor protein of muscle thick filaments
2	77254	*Yif1b*	3.40	*Yip1 interacting factor homolog B (S. cerevisiae)*	Unknown
3	16783	*Lamp1*	3.39	*Lysosomal-associated membrane protein 1*	Presents carbohydrate ligands to selectins
4	18453	*P4hb*	3.31	*Prolyl 4-hydroxylase, beta polypeptide*	Catalyzes the formation, breakage and rearrangement of disulfide bonds
5	328234	*Rnf182*	3.20	*Ring finger protein 182*	E3 ubiquitin-protein ligase
6	66184	*Rps4y2*	3.09	*Ribosomal protein S4, Y-linked 2*	Unknown
7	14230	*Fkbp10*	3.08	*FK506 binding protein 10*	PPIases accelerate the folding of proteins during protein synthesis
8	11803	*Aplp1*	3.05	*Amyloid beta (A4) precursor-like protein 1*	May play a role in postsynaptic function
9	72053	*2010008E23rik*	3.02	*Transmembrane and ubiquitin-like domain containing 2*	Unknown

Table S3. *Cont.*

No.	Gene ID	Gene Symbol	E12.5 Proximal/E13.5 Carpal Ratio of Expression	Gene Name	Function
10	216964	*2410019G02Rik*	2.98	*Transformation related protein 53 inducible protein 13*	Unknown
11	235504	*Slc17a5*	2.97	*Solute carrier family 17 (anion/sugar transporter), member 5*	Primary solute translocator for anionic substances
12	434204	*Whdc1*	2.95	*WAS protein homolog associated with actin, golgi membranes and microtubules*	Acts as a nucleation-promoting factor (NPF) that stimulates Arp2/3-mediated actin
13	107951	*Cdk9*	2.88	*Cyclin-dependent kinase 9 (CDC2-related kinase)*	Protein kinase involved in the regulation of transcription.
14	107729	*Ubg*	2.83	*Ubiquitin, beta-galactosidase related*	Unknown
15	16010	*Igfbp4*	2.81	*Insulin-like growth factor binding protein 4*	Prolong the half-life of the IGFs and have been shown to either inhibit or stimulate the growth
16	224650	*Anks1*	2.78	*Ankyrin repeat and SAM domain containing 1*	Regulator of different signaling pathways. Regulates EPHA8 receptor tyrosine kinase signaling
17	225849	*Ppp2r5b*	2.78	*Protein phosphatase 2, regulatory subunit B (B56), beta isoform*	Unknown
18	8665	*Eif3f*	2.53	*Eukaryotic translation initiation factor 3, subunit F*	Component of the eIF-3 complex, required for several steps in the initiation of protein synthesis
19	399673	*Tdpoz2*	2.48	*TD and POZ domain containing 2*	TDPOZ, a family of bipartite animal and plant proteins that contain the TRAF (TD) and POZ/BTB domains.
20	666676	*LOC666676*	2.48	*Predicted gene 8230*	Unknown
21	109205	*5330439J01Rik*	2.41	*Sine oculis-binding protein homolog (Drosophila)*	Strong expression in the limbic system at the time interval of active synaptogenesis.
22	66043	*Atp5d*	2.40	*ATP synthase, H+ transporting, mitochondrial F1 complex, delta subunit*	Produces ATP from ADP in the presence of a proton gradient across the membrane.
23	21804	*Tgfb1l1*	2.39	*Transforming growth factor beta 1 induced transcript 1*	A molecular adapter coordinating multiple protein–protein interactions at the focal adhesion complex

Table S3. *Cont.*

No.	Gene ID	Gene Symbol	E12.5 Proximal/E13.5 Carpal Ratio of Expression	Gene Name	Function
24	19223	*Ptgis*	2.36	*Prostaglandin I2 (prostacyclin) synthase*	Catalyzes the isomerization of prostaglandin H2 to prostaglandin I2
25	70020	*Znhit4*	2.35	*INO80 complex subunit B*	Involved in transcriptional regulation, DNA replication and probably DNA repair
26	12522	*Cd83*	2.34	*CD83 antigen*	Associates with CD4 or CD8 and delivers costimulatory signals for the TCR/CD3 pathway
27	217351	*9930033H14Rik*	2.34	*Trinucleotide repeat containing 6C*	Plays a role in RNA-mediated gene silencing by micro-RNAs (miRNAs)
28	108013	*A230070D14Rik*	2.33	*CUGBP, Elav-like family member 4*	RNA-binding protein implicated in the regulation of pre-mRNA alternative splicing
29	230073	*Ddx58*	2.31	*DEAD (Asp-Glu-Ala-Asp) box polypeptide 58*	Innate immune receptor which acts as a cytoplasmic sensor of viral nucleic acids
30	94230	*Cpsf1*	2.28	*Cleavage and polyadenylation specific factor 1*	Pre-mRNA 3'-end formation, recognizing the AAUAAA signal sequence
31	217030	*Ap1gbp1*	2.27	*Synergin, gamma*	Endocytosis and/or membrane trafficking at the trans-Golgi network
32	57773	*Wdr4*	2.26	*WD repeat domain 4*	Required for the formation of N(7)-methylguanine at position 46 (m7G46) in tRNA
33	66085	*Eif3s5*	2.26	*Eukaryotic translation initiation factor 3, subunit F*	Component of the eukaryotic translation initiation factor 3 (eIF-3) complex
34	19727	*Rfxank*	2.25	*regulatory factor X-associated ankyrin-containing protein*	Activates transcription from class II MHC promoters
35	258293	*Olfr437*	2.24	*Olfactory receptor 437*	Interact with odorant molecules in the nose
36	29862	*Ninj2*	2.21	*Ninjurin 2*	Homophilic cell adhesion molecule that promotes axonal growth
37	22174	*Tyro3*	2.20	*TYRO3 protein tyrosine kinase 3*	Receptor tyrosine kinase that transduces signals from the extracellular matrix into the cytoplasm

Table S3. *Cont.*

No.	Gene ID	Gene Symbol	E12.5 Proximal/E13.5 Carpal Ratio of Expression	Gene Name	Function
38	12705	*Cited1*	2.18	*Cbp/p300-interacting transactivator with Glu/Asp-rich carboxy-terminal domain 1*	Transcriptional coactivator of the p300/CBP-mediated trancription complex
39	14950	*H13*	2.18	*Histocompatibility 13*	Catalyzes intramembrane proteolysis of some signal peptides after they have been cleaved
40	56212	*Rhog*	2.15	*Ras homolog gene family, member G*	Required for the formation of membrane ruffles during macropinocytosis
41	13057	*Cyba*	2.11	*Cytochrome b-245, alpha polypeptide*	Critical component of the membrane-bound oxidase of phagocytes that generates superoxide
42	13650	*Rhbdf1*	2.10	*Rhomboid family 1 (Drosophila)*	Regulates the secretion of several ligands of the epidermal growth factor receptor
43	13008	*Csrp2*	2.09	*Cysteine and glycine-rich protein 2*	CRP2 acted as a potent transcription coadaptor that remodeled silent cardiac myocyte chromatin
44	57436	*Gabarapl1*	2.09	*Gamma-aminobutyric acid (GABA) A receptor-associated protein-like 1*	Negatively regulates Wnt/beta-catenin signaling by mediating Dvl2 degradation
45	100113398	*A430024h01rik*	2.09	*Adenosine deaminase, tRNA-specific 3, TAD2 homolog (S. cerevisiae)*	Deamination of adenosine-34 to inosine in many tRNAs as a regulatory subunit Potential
46	51792	*Ppp2r1a*	2.08	*Protein phosphatase 2 (formerly 2A), regulatory subunit A (PR 65), alpha isoform*	Required for proper chromosome segregation and for centromeric localization of SGOL1 in mitosis
47	223776	*1300018J18Rik*	2.08	*RIKEN cDNA 1300018J18 gene*	A selenoprotein, which contains a selenocysteine (Sec) residue at its active site
48	15488	*Hsd17b4*	2.05	*Hydroxysteroid (17-beta) dehydrogenase 4*	Bifunctional enzyme acting on the peroxisomal beta-oxidation pathway for fatty acids
49	106068	*Slc45a4*	2.05	*Solute carrier family 45, member 4*	Unknown
50	58202	*Cobra1*	2.04	*Cofactor of BRCA1*	Essential to the NELF complex, negatively regulates the elongation of transcription by RNA polymerase II

Table S3. *Cont.*

No.	Gene ID	Gene Symbol	E12.5 Proximal/E13.5 Carpal Ratio of Expression	Gene Name	Function
51	12826	*Col4a1*	2.02	*Collagen, type IV, alpha 1*	Type IV collagen is the major structural component of glomerular basement membranes
52	22384	*Eif4h*	2.00	*Eukaryotic translation initiation factor 4H*	Stimulates the RNA helicase activity of EIF4A in the translation initiation complex
53	17149	*Magoh*	0.49	*Mago-nashi homolog, proliferation-associated (Drosophila)*	Mouse magoh is involved in cyclin-dependent kinase regulation.
54	326619	*Hist1h4a*	0.49	*Histone cluster 1, H4a*	Core component of nucleosome. Nucleosomes wrap and compact DNA into chromatin
55	67154	*Mtdh*	0.48	*Metadherin*	Downregulates SLC1A2/EAAT2 promoter activity when expressed ectopically
56	67713	*Dnajc19*	0.48	*DnaJ (Hsp40) homolog, subfamily C, member 19*	Probable component of a complex required for the translocation of transit peptide-containing proteins
57	68735	*Mrps18c*	0.48	*Mitochondrial ribosomal protein S18C*	Unknown
58	70257	*2010107E04Rik*	0.48	*RIKEN cDNA 2010107E04 gene*	Unknown
59	109672	*Cyb5*	0.48	*Cytochrome b-5*	Cytochrome b5 is a membrane bound hemoprotein which function as an electron carrier
60	225058	*LOC225058*	0.48	*Predicted gene 4832*	Unknown
61	319180	*Hist1h2bf*	0.48	*Histone cluster 1, H2bf*	Core component of nucleosome
62	69010	*Anapc13*	0.46	*Anaphase promoting complex subunit 13*	Component of the anaphase promoting complex/cyclosome
63	78651	*Lsm6*	0.46	*LSM6 homolog, U6 small nuclear RNA associated (S. cerevisiae)*	Component of LSm complexes, involved in RNA processing and may function in a chaperone-like manner

Table S3. *Cont.*

No.	Gene ID	Gene Symbol	E12.5 Proximal/E13.5 Carpal Ratio of Expression	Gene Name	Function
64	66915	*Myevo2*	0.45	*Myeloma overexpressed 2*	Unknown
65	69702	*Ndufaf1*	0.45	*NADH dehydrogenase (ubiquinone) 1 alpha subcomplex, assembly factor 1*	Chaperone protein involved in the assembly of the mitochondrial NADH:ubiquinone oxidoreductase complex
66	72657	*2700094K13Rik*	0.45	*RIKEN cDNA 2700094K13 gene*	A selenoprotein, which contains a selenocysteine (Sec) residue at its active site.
67	66286	*Sec11c*	0.44	*SEC11 homolog C (S. cerevisiae)*	Component of the microsomal signal peptidase complex which removes signal peptides from nascent proteins
68	54405	*Ndufa1*	0.43	*NADH dehydrogenase (ubiquinone) 1 alpha subcomplex, 1*	Accessory subunit of the mitochondrial membrane respiratory chain NADH dehydrogenase
69	100043304	*LOC100047905*	0.43	*S-adenosylmethionine decarboxylase, pseudogene 3*	Unknown
70	17709	*Mt-co2*	0.42	*Cytochrome c oxidase subunit II*	component of the respiratory chain Subunit 2 transfers the electrons from cytochrome c
71	668171	*Zxda*	0.42	*zinc finger, X-linked, duplicated A*	Cooperates with CIITA to promote transcription of MHC class I and MHC class II genes
72	8626578	*Nola3*	0.41	*H/ACA RNP complex subunit 3*	Unknown
73	14156	*Fen1*	0.40	*Flap structure specific endonuclease 1*	FEN1 has roles in DNA repair, as well as in DNA replication
74	15387	*Hnrpk*	0.40	*Heterogeneous nuclear ribonucleoprotein K*	One of the major pre-mRNA-binding proteins
75	27356	*Insl6*	0.40	*Insulin-like 6*	Most abundantly expressed in tissues rich in highly ciliated cells and is predicted to be important to cilia
76	15126	*Hba-x*	0.38	*Hemoglobin X, alpha-like embryonic chain in Hba complex*	The zeta chain is an alpha-type chain of mammalian embryonic hemoglobin
77	15528	*Hspe1*	0.38	*Heat shock protein 1 (chaperonin 10)*	Heat shock protein 10 inhibits lipopolysaccharide-induced inflammatory mediator production

Table S3. *Cont.*

No.	Gene ID	Gene Symbol	E12.5 Proximal/E13.5 Carpal Ratio of Expression	Gene Name	Function
78	54367	*Zfp326*	0.38	*Zinc finger protein 326*	Core component of the DBIRD complex, a multiprotein complex
79	67371	*2410016F19Rik*	0.38	*General transcription factor IIIC, polypeptide 6, alpha*	Involved in RNA polymerase III-mediated transcription
80	67899	*2010110K16Rik*	0.38	*COX assembly mitochondrial protein homolog (S. cerevisiae)*	Required for mitochondrial cytochrome c oxidase (COX) assembly and respiration. Binds copper
81	73736	*1110008B24Rik*	0.38	*FCF1 small subunit (SSU) processome component homolog (S. cerevisiae)*	Essential protein involved in pre-rRNA processing and 40S ribosomal subunit assembly By similarity
82	76915	*Mnd1*	0.38	*Meiotic nuclear divisions 1 homolog (S. cerevisiae)*	Required for proper homologous chromosome pairing and efficient cross-over and intragenic recombination during meiosis
83	319158	*Hist1h4i*	0.37	*histone cluster 1, H4i*	Core component of nucleosome. Nucleosomes wrap and compact DNA into chromatin
84	26450	*Rbbp9*	0.35	*Retinoblastoma binding protein 9*	RBBP9 is expression inversely correlated with RB phosphorylation
85	20533	*Slc4a1*	0.34	*Solute carrier family 4 (anion exchanger), member 1*	Band 3 is the major integral glycoprotein of the erythrocyte membrane
86	17972	*Ncf4*	0.32	*Neutrophil cytosolic factor 4*	Component of the NADPH-oxidase, a multicomponent enzyme system responsible for the oxidative burst
87	109978	*Art4*	0.25	*ADP-ribosyltransferase 4*	May be part of a novel protein family with an important role in regulating cell function
88	170812	*Eraf*	0.23	*Alpha hemoglobin stabilizing protein*	Chaperone for free alpha-hemoglobin
89	100000000	*LOC100040413*	0.22	*Predicted gene 10075*	Unknown
90	11806	*Apoa1*	0.21	*Apolipoprotein A-I*	Reverse transport of cholesterol from tissues to the liver for excretion
91	54419	*Cldn6*	0.14	*Claudin 6*	Plays a major role in tight junction-specific obliteration of the intercellular space

Table S4. Differential gene expression between the proximal region of E12.5 handplate and the metacarpal-phalange region of E13.5 mouse handplate.

No.	Gene ID	Gene Symbol	E12.5 Proximal/E13.5 Metacarpal-Phalange Ratio of Expression	Gene Name	Function
1	17885	*Myh8*	7.06	*Myosin, heavy polypeptide 8, skeletal muscle, perinatal*	Motor protein of muscle thick filaments
2	224650	*Anks1*	3.55	*Ankyrin repeat and SAM domain containing 1*	Regulator of different signaling pathways
3	66184	*Rps4y2*	3.43	*Ribosomal protein S4, Y-linked 2*	Unknown
4	399673	*Tdpoz2*	3.39	*TD and POZ domain containing 2*	TDPOZ, a family of bipartite animal and plant proteins that contain the TRAF (TD) and POZ/BTB domains
5	434204	*Whdc1*	3.3	*WAS protein homolog associated with actin, golgi membranes and microtubules*	Acts as a nucleation-promoting factor (NPF) that stimulates Arp2/3-mediated actin polymerization
6	217030	*Synrg*	3.15	*Synergin, gamma*	May play a role in endocytosis and/or membrane trafficking at the trans-Golgi network
7	103172	*Ndg2*	3.07	*Coiled-coil-helix-coiled-coil-helix domain containing 10*	Unknown
8	21847	*Klf10*	2.99	*Kruppel-like factor 10*	Transcriptional repressor involved in the regulation of cell growth. Inhibits cell growth
9	71913	*Tmem79*	2.85	*Transmembrane protein 79*	Unknown
10	11472	*Actn2*	2.8	*Actinin alpha 2*	Protein anchors actin to a variety of intracellular structures. This is a bundling protein
11	16783	*Lamp1*	2.8	*Lysosomal-associated membrane protein 1*	Presents carbohydrate ligands to selectins. Also implicated in tumor cell metastasis
12	17896	*Myl4*	2.8	*Myosin, light polypeptide 4*	Regulatory light chain of myosin. Does not bind calcium
13	15129	*Hbb-b1*	2.77	*Hemoglobin, beta adult major chain*	Involved in oxygen transport from the lung to the various peripheral tissues.
14	20739	*Spna1*	2.72	*Spna1 spectrin alpha 1*	Major constituent of the cytoskeletal network underlying the erythrocyte plasma membrane
15	12522	*Cd83*	2.65	*CD83 antigen*	Associates with CD4 or CD8 and delivers costimulatory signals for the TCR/CD3 pathway

Table S4. *Cont.*

No.	Gene ID	Gene Symbol	E12.5 Proximal/E13.5 Metacarpal-Phalange Ratio of Expression	Gene Name	Function
16	58805	*Wbscr14*	2.63	*MLX interacting protein-like*	Transcriptional repressor
17	68895	*Rasl11a*	2.62	*RAS-like, family 11, member A*	Regulator of rDNA transcription. Acts in cooperation UBF/UBTF and positively regulates RNA polymerase I
18	235504	*Slc17a5*	2.6	*Solute carrier family 17 (anion/sugar transporter), member 5*	Solute translocator for anionic substances
19	217365	*Nploc4*	2.55	*Nuclear protein localization 4 homolog (S. cerevisiae)*	Necessary for the export of misfolded proteins from the ER to the cytoplasm
20	19659	*Rbp1*	2.51	*Retinol binding protein 1, cellular*	Intracellular transport of retinol
21	68204	*2900060B14Rik*	2.47	*RIKEN cDNA 2900060B14 gene*	Unknown
22	76367	*Trp53rk*	2.46	*Transformation related protein 53 regulating kinase*	Protein kinase that phosphorylates "Ser-15" of p53/TP53 protein and may participate in its activation
23	14941	*Ubg*	2.45	*Ubiquitin, beta-galactosidase related*	Unknown
24	14941	*Gzmd*	2.45	*Granzyme D*	Unknown
25	80880	*Kank3*	2.44	*KN motif and ankyrin repeat domains 3*	Unknown
26	94230	*Cpsf1*	2.43	*Cleavage and polyadenylation specific factor 1*	Component of the cleavage and polyadenylation specificity factor (CPSF) complex
27	21380	*Tbx1*	2.41	*T-box 1*	Key regulatory gene in development, is highly expressed in hair follicle (HF) stem cells in adult mice
28	69719	*Cad*	2.4	*Carbamoyl-phosphate synthetase 2, aspartate transcarbamylase, and dihydroorotase*	Unknown
29	13139	*Dgka*	2.38	*Diacylglycerol kinase, alpha*	Converts the second messenger diacylglycerol into phosphatidate
30	666676	*LOC666676*	2.36	*Predicted gene 8230*	Unknown
31	52846	*D1bwg0212e*	2.33	*DNA segment, Chr 1, Brigham & Women's Genetics 0212 expressed*	Unknown
32	26383	*Fto*	2.33	*Fat mass and obesity associated*	Contributes to the regulation of body size and body fat accumulation

Table S4. *Cont.*

No.	Gene ID	Gene Symbol	E12.5 Proximal/E13.5 Metacarpal-Phalange Ratio of Expression	Gene Name	Function
33	29862	*Ninj2*	2.32	*Ninjurin 2*	Homophilic cell adhesion molecule that promotes axonal growth
34	12521	*Cd82*	2.31	*CD82 antigen*	Associates with CD4 or CD8 and delivers costimulatory signals for the TCR/CD3 pathway
35	20393	*Sgk1*	2.31	*Serum/glucocorticoid regulated kinase 1*	Regulation of a wide variety of ion channels, membrane transporters, cellular enzymes, transcription factors, neuronal excitability, cell growth, proliferation, survival, migration and apoptosis
36	545253	*Eg545253*	2.29	*predicted gene 5820*	Unknown
37	66043	*Atp5d*	2.28	*ATP synthase, H+ transporting, mitochondrial F1 complex, delta subunit*	Produces ATP from ADP in the presence of a proton gradient across the membrane which is generated by electron transport
38	224897	*Dpp9*	2.28	*Dipeptidylpeptidase 9*	Dipeptidyl peptidase
39	11629	*Aif1*	2.26	*Allograft inflammatory factor 1*	Enhances the actin-bundling activity of LCP1
40	72454	*Ccdc71*	2.25	*coiled-coil domain containing 71*	Unknown
41	54473	*Tollip*	2.24	*Toll interacting protein*	Recruits IRAK1 to the IL-1 receptor complex
42	18453	*P4hb*	2.23	*Prolyl 4-hydroxylase, beta polypeptide*	This multifunctional protein catalyzes the formation, breakage and rearrangement of disulfide bonds
43	109205	*5330439J01RIK/Sobp*	2.23	*Sine oculis-binding protein homolog (Drosophila)*	Implicated in development of the cochlea
44	223870	*Senp1*	2.22	*SUMO1/sentrin specific peptidase 1*	Protease that catalyzes two essential functions in the SUMO pathway
45	70335	*Reep6*	2.21	*Receptor accessory protein 6*	May enhance the cell surface expression of odorant receptors
46	13057	*Cyba*	2.19	*Cytochrome b-245, alpha polypeptide*	Critical component of the membrane-bound oxidase of phagocytes that generates superoxide
47	69009	*Thap7*	2.18	*THAP domain containing 7*	Chromatin-associated, histone tail-binding protein that represses transcription
48	107951	*Cdk9*	2.18	*Cyclin-dependent kinase 9 (CDC2-related kinase)*	Protein kinase involved in the regulation of transcription

Table S4. *Cont.*

No.	Gene ID	Gene Symbol	E12.5 Proximal/E13.5 Metacarpal-Phalange Ratio of Expression	Gene Name	Function
49	11927	*Atox1*	2.15	*ATX1 (antioxidant protein 1) homolog 1 (yeast)*	Could bind and deliver cytosolic copper to the copper ATPase proteins
50	17748	*Mt1*	2.15	*Metallothionein 1*	These proteins are transcriptionally regulated by both heavy metals and glucocorticoids
51	70020	*Znhit4*	2.15	*INO80 complex subunit B*	Proposed core component of the chromatin remodeling INO80 complex
52	211499	*Tmem87A*	2.13	*transmembrane protein 87A*	Negative regulation of phosphatase activity Inferred from sequence or structural similarity
53	12826	*Col4a1*	2.1	*collagen, type IV, alpha 1*	Major structural component of glomerular basement membranes
54	77254	*Yif1b*	2.09	*Yip1 interacting factor homolog B (S. cerevisiae)*	Unknown
55	21849	*Trim28*	2.08	*Tripartite motif-containing 28*	Nuclear corepressor for KRAB domain-containing zinc finger proteins (KRAB-ZFPs)
56	20856	*Stc2*	2.06	*Stanniocalcin 2*	Has an anti-hypocalcemic action on calcium and phosphate homeostasis
57	56212	*Rhog*	2.06	*Ras homolog gene family, member G*	Required for the formation of membrane ruffles during macropinocytosis
58	230766	*Bc030183*	2.06	*Family with sequence similarity 167, member B*	Unknown
59	320634	*9530014D17Rik*	2.06	*Oculocerebrorenal syndrome of Lowe*	Unknown
60	192191	*Med9*	2.05	*Mediator of RNA polymerase II transcription, subunit 9 homolog (yeast)*	A coactivator involved in the regulated transcription of nearly all RNA polymerase II-dependent genes
61	14230	*Fkbp10*	2.04	*FK506 binding protein 10*	PPIases accelerate the folding of proteins during protein synthesis
62	16010	*Igfbp4*	2.03	*Insulin-like growth factor binding protein 4*	Either inhibit or stimulate the growth promoting effects of the IGFs on cell culture
63	100113398	*A430024h01Rik/Adat3*	2.03	*Adenosine deaminase, tRNA-specific 3, TAD2 homolog (S. cerevisiae)*	May be involved in deamination of adenosine-34 to inosine in many tRNAs as a regulatory subunit
64	385063	*LOC385063*	2.03	*Hippocalcin-like protein 1-like*	Unknown
65	66839	*0610009O20Rik*	2.02	*RIKEN cDNA 0610009O20 gene*	Unknown

Table S4. *Cont.*

No.	Gene ID	Gene Symbol	E12.5 Proximal/E13.5 Metacarpal-Phalange Ratio of Expression	Gene Name	Function
66	72357	*2210016L21Rik*	2.02	*RIKEN cDNA 2210016L21 gene*	Unknown
67	72238	*Tbc1d5*	2.01	*TBC1 domain family, member 5*	May act as a GTPase-activating protein for Rab family protein(s)
68	108657	*Rnpepl1*	2.01	*Arginyl aminopeptidase (aminopeptidase B)-like 1*	Unknown
69	11461	*Actb*	2	*Actin, beta*	Highly conserved proteins that are involved in various types of cell motility
70	13384	*Mpp3*	0.49	*Membrane protein, palmitoylated 3 (MAGUK p55 subfamily member 3)*	Unknown
71	14734	*Gpc3*	0.48	*Gypican 3*	GPC3 acts as a negative regulator of Hedgehog signaling during mammalian development
72	230726	*Rhbdl2*	0.48	*Rhomboid, veinlet-like 2 (Drosophila)*	Intramembrane proteolysis and the subsequent release of functional polypeptides from their membrane anchors
73	15980	*Ifngr2*	0.47	*Interferon gamma receptor 2*	A pleiotropic cytokine that plays an important role in many inflammatory processes
74	54419	*Cldn6*	0.47	*Claudin 6*	Plays a major role in tight junction-specific obliteration of the intercellular space
75	668171	*Zxda*	0.47	*zinc finger, X-linked, duplicated A*	Cooperates with CIITA to promote transcription of MHC class I and MHC class II genes
76	109129	*2010311D03Rik*	0.46	*Methylmalonic aciduria (cobalamin deficiency) cblD type, with homocystinuria*	During organogenesis Mmachc and Mmadhc may interact in only a subset of cells
77	54367	*Zfp326*	0.45	*Zinc finger protein 326*	Integrates transcript elongation with the regulation of alternative splicing
78	16876	*Lhx9*	0.44	*LIM homeobox protein 9*	Gonadal development
79	66286	*Sec11c*	0.41	*SEC11 homolog C (S. cerevisiae)*	Removes signal peptides from nascent proteins as they are translocated into the lumen of the ER

Table S4. *Cont.*

No.	Gene ID	Gene Symbol	E12.5 Proximal/E13.5 Metacarpal-Phalange Ratio of Expression	Gene Name	Function
80	319158	*Hist1h4i*	0.41	*Histone cluster 1, H4i*	Core component of nucleosome. Nucleosomes wrap and compact DNA into chromatin
81	17972	*Ncf4*	0.4	*Neutrophil cytosolic factor 4*	Component of the NADPH-oxidase
82	244710	*LOC244710*	0.4	*Predicted gene 4977*	Unknown Function
83	59003	*Maea*	0.39	*Macrophage erythroblast attacher*	Plays a role in erythroblast enucleation and in the development of the mature macrophages
84	270665	*LOC270665*	0.39	*Predicted gene 5060*	Unknown
85	26450	*Rbbp9*	0.37	*Retinoblastoma binding protein 9*	RBBP9 is expression inversely correlated with RB phosphorylation
86	17709	*Mt-co2*	0.36	*Cytochrome c oxidase subunit II*	Cytochrome c oxidase is the component of the respiratory chain. Subunit 2 transfers the electrons from cytochrome c
87	55936	*Ctps2*	0.3	*Cytidine 5'-triphosphate synthase 2*	Constitutes the rate-limiting enzyme in the synthesis of cytosine nucleotides
88	100715	*Papd4*	0.28	*PAP associated domain containing 4*	Cytoplasmic poly(A) RNA polymerase that adds successive AMP monomers to the 3'-end of specific RNAs, forming a poly(A) tail
89	15387	*Hnrpk*	0.23	*Heterogeneous nuclear ribonucleoprotein K*	. Binds tenaciously to poly(C) sequences. Likely to play a role in the nuclear metabolism of hnRNAs
90	11806	*Apoa1*	0.22	*Apolipoprotein A-I*	Participates in the reverse transport of cholesterol from tissues to the liver for excretion
91	100040413	*LOC100040413*	0.19	*Predicted gene 10075*	Unknown

© 2014 by the authors; licensee MDPI, Basel, Switzerland. This article is an open access article distributed under the terms and conditions of the Creative Commons Attribution license (http://creativecommons.org/licenses/by/3.0/).
